# Hyperspectral Imaging with Machine Learning Approaches for Assessing Soluble Solids Content of Tribute Citru

**DOI:** 10.3390/foods12020247

**Published:** 2023-01-05

**Authors:** Cheng Li, Mengyu He, Zeyi Cai, Hengnian Qi, Jianhong Zhang, Chu Zhang

**Affiliations:** School of Information Engineering, Huzhou University, Huzhou 313000, China

**Keywords:** hyperspectral images, soluble solids content, machine learning, sampling sides, data fusion

## Abstract

Tribute Citru is a natural citrus hybrid with plenty of vitamins and nutrients. Fruits’ soluble solids content (SSC) is a critical quality index. This study used hyperspectral imaging at two spectral ranges (400–1000 nm and 900–1700 nm) to determine SSC in Tribute Citru. Partial least squares regression (PLSR) and support vector regression (SVR) models were established in order to determine SSC using the spectral information of the calyx and blossom ends. The average spectra of both ends as well as their fusion was studied. The successive projections algorithm (SPA) and the correlation coefficient analysis (CCA) were used to examine the differences in characteristic wavelengths between the two ends. Most models achieved performances with the correlation coefficient of the training, validation, and testing sets over 0.6. Results showed that differences in the performances among the models using the one-sided and two-sided spectral information. No particular regulation could be found for the differences in model performances and characteristic wavelengths. The results illustrated that the sampling side was an influencing factor but not the determinant factor for SSC determination. These results would help with the development of real-world applications for citrus quality inspection without concerning the sampling sides and the spectral ranges.

## 1. Introduction

China is now the country with the largest production of citrus. Citrus is a popular fruit not only for its unique taste but also for its high nutritional content. There are many excellent cultivars of citrus throughout the world. As one of them, Tribute Citru, also known as emperor citrus or Gong citrus, is a kind of citrus with high contents of carbohydrates, fiber, protein, calcium, etc. [1].

The soluble solids content (SSC) is a crucial indicator for evaluating the quality of Tribute Citru. SSC is the primary and most important quality parameter of concern to consumers. The common method of measuring SSC is to use a refractometer to measure the squeezed juice, which is time-consuming and damaging to the sample. Moreover, it cannot meet the demands for large-scale non-destructive measurement. Thus, rapid and non-destructive analytical techniques are needed. 

Visible/near-infrared spectroscopy is a rapid and non-destructive technique that does not produce chemical contamination or damage to the sample(s) and has been applied in detecting fruit SSC [2,3,4]. Hyperspectral imaging technology combines imaging and spectroscopy technology, which can obtain spatial information at continuous spectral bands with high spectral resolution. Many research papers have been published based upon using hyperspectral imaging technology to detect SSC in fruits [5,6,7,8,9].

There are variations to the physiochemical characteristics within different parts of the fruit. VIS/NIR spectroscopy conducts point scan. When collecting spectral information of fruits, the different sampling points along with the equator or other representative regions were generally collected [10,11,12,13]. Some studies have also discussed the influence of spectra acquisition from different areas for SSC measurement [14,15,16].

The impact of variation within the samples should be considered in order to create a more accurate and robust SSC evaluation method. Hyperspectral imaging (HSI) can acquire the image of one side of the sample, covering the variations within the side of the sample. However, most studies acquired hyperspectral images of only one side of the samples [5,6,7]. Rare studies have considered using different sides of the fruits for SSC determination [8,9,17].

The SSC values were generally obtained to establish prediction models by averaging the measured values of different sample parts. The spectrum of each sample was also averaged by the spectra of different scanning points. Thus, it would be better if both the SSC value and the spectrum represent the corresponding sample. Given that different areas have both variations and similarities, it was worth investigating whether measuring both sides of samples for HSI was necessary. On the other hand, researchers have shown that hyperspectral imaging at different spectral ranges could be used to determine the SSC of fruits. The spectral ranges were also investigated to explore the influence of sampling sides. 

In this study, hyperspectral imaging was used to determine SSC in Tribute Citru. The specific objectives were to (1) explore and compare the performances of hyperspectral imaging for SSC determination at two different spectral ranges (400–1000 nm and 900–1700 nm); (2) explore and compare the performances of sampling sides (the calyx end and the blossom end) for SSC determination; (3) explore the performances for SSC determination using the average spectra of the two sides and fused spectra of the two sides; (4) explore the characteristic wavelengths for SSC determination using the spectra of two sides to evaluate the performance consistency by the two sides.

## 2. Materials and Methods

### 2.1. Sample Preparation

**Figure 1:**
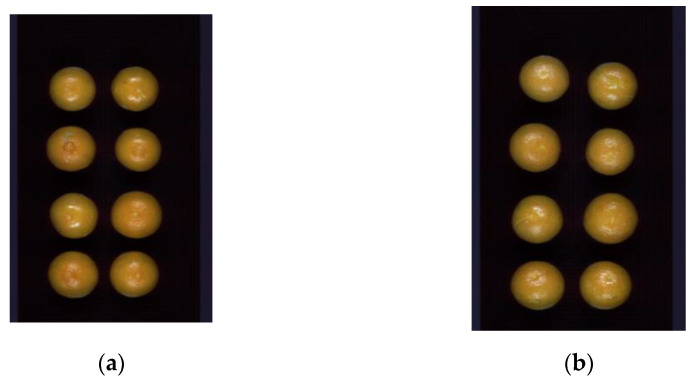
RGB images of Tribute Citru. (**a**): the fruit blossom end; (**b**) the fruit calyx end.

Tribute Citru fruits were purchased from a local orchard in Liuzhou, Guangxi Province, China. A total of 458 samples of Tribute Citru fruits with similar sizes and no apparent damage on the surface were harvested on 22 November 2021. Before image acquisition, the fruits were stored at transported for four days and stored in the experimental room at a relatively stable temperature (15 ± 1 ℃) for three days. All the samples were numbered sequentially and awaited the acquisition of hyperspectral images and subsequent SSC measurement. Figure 1 shows the RGB (red, green, and blue) images of citrus taken by the hyperspectral imaging system. The image of the fruit blossom end and the calyx end are shown in Figure 1a,b, respectively. 

### 2.2. SSC Measurement

The experimental arrangement for measuring SSC of Tribute Citru included a WIGGENS BR0035 Digital Brix Refractometer (WIGGENS Technology Ltd., Beijing, China) with an accuracy of 0.1 and °Brix range of 0–35%. For each of the 458 samples, the fruit was cut into three pieces by the axis of the calyx and the blossom. Each piece was squeezed, and the juice was used to measure the SSC value. To avoid contamination, the refractometer was washed with distilled water after each measurement and dried with clean paper before the next reading. The average SSC value of the three pieces was used as the SSC of the sample.

### 2.3. Hyperspectral Imaging Systems

In this study, hyperspectral imaging systems at two spectral ranges were used for image acquisition of citrus. The two hyperspectral imaging systems differed in the hyperspectral camera. One hyperspectral camera was FX10 (Spectral Imaging Ltd., Oulu, Finland) with a spectral range of 400–1000 nm (VIS/NIR), and the other hyperspectral camera was FX17 (Spectral Imaging Ltd., Oulu, Finland) with a spectral range of 900–1700 nm. The two hyperspectral imaging systems shared the same light source (covering the spectral range of 35–2500 nm), moving platform, computer, and image acquisition software. First, the hyperspectral images of all the samples were acquired by FX10 camera. Next, the camera was changed to FX17 without changing the other hardware, and the hyperspectral images of the samples were then acquired by FX17 camera. The hyperspectral images were generated by pushbroom imaging (line scanning).

### 2.4. Hyperspectral Image Acquisition and Spectra Extraction

#### 2.4.1. Hyperspectral Image Acquisition

The samples were placed on the mobile platform in sequence for hyperspectral image acquisition, as shown in Figure 1. The system parameters were adjusted for hyperspectral image acquisition. For FX10, the moving speed of the mobile platform was 17 mm/s, and the exposure time was set as 13.59 ms. For FX17, the moving speed of the mobile platform was set as 25 mm/s, and the exposure time was set as 8.26 ms. The difference in speed was because the imaging time of the two cameras was inconsistent. The hyperspectral camera will scan the calyx and blossom ends of Tribute Citru as side A and side B samples, respectively.

#### 2.4.2. Spectra Extraction

Eight or fewer fruits were acquired in one hyperspectral image. Each fruit within the hyperspectral image was first cut as sub-hyperspectral images. The ENVI 4.7 software (ITT, Visual Information Solutions, Boulder, CO, USA) was used to cut each of the 458 samples from the original hyperspectral images, after which each sub-hyperspectral image contained only one fruit. The entire fruit region was defined as the region of interest (ROI), and the average spectrum of all pixel-wise spectra within the ROI was extracted.

Due to the noise at the beginning and end of the original spectra, the wavelength range of 487–974 nm was selected for FX10 and 933–1690 nm for FX17 of each sample. To eliminate the background interference and reduce the sample shape influence, wavelet transformation and area normalization preprocessing algorithms were used to preprocess the spectra. The spectral dataset of the fruit calyx end was named SP-A, and the spectral dataset of the fruit blossom end was named SP-B. By measuring the two sides of the samples, information of the entire fruit could be obtained, and more variations could be covered.

### 2.5. Data Analysis Methods

#### 2.5.1. Regression Models

Support vector regression (SVR) is an important applied branch of support vector machine (SVM), a supervised learning algorithm for predicting discrete values [18]. The goal of SVR is to construct a hyperplane to fit the samples to minimize the overall distance between all samples and the hyperplane. SVR assumes that an error between the output value and the predicted value can be tolerated, and only the loss is calculated when the absolute value of the difference between the two is larger than the error. Therefore, SVR has a certain degree of tolerance to outliers, robustness, and excellent generalization ability. SVR can solve both linear and non-linear issues. For non-linear problems, kernel functions are crucial to map the original data into high-dimensional spaces. In this study, the radial basis function (RBF) was used as the kernel function. The regularization parameters and kernel function coefficients were optimized by the grid-search method in the range of [10^−6^, 10^6^].

Partial least squares regression (PLSR) is used to seek the underlying linear relationship between two matrices (independent matrix X and dependent matrix Y), which is a hidden variable approach to modeling the covariance structure in these two vector spaces. In order to explain the multidimensional direction with the biggest variance in the Y-space, the PLSR model tries to identify the multidimensional direction of the X-space [19]. PLSR decomposes both variables X and Y to extract factors, then the latent factors are arranged in descending order of their correlation. In contrast to conventional mulblossomle linear regression (MLR) models, PLSR can be constructed with mulblossomle correlated independent variables. The model can also be built if the number of samples is less than that of variables.

#### 2.5.2. Wavelength Selection Methods

Successive projections algorithm (SPA) is a forward feature variable selection method. By projecting wavelengths onto other wavelengths, SPA compares the projection vectors, and the wavelength with the largest projection vector is selected into the candidate subset of characteristic wavelengths. The regression model is then used to evaluate the performances of different subsets. Therefore, SPA selects the combination of variables that contains the least redundant information and the least covariance [20].

Correlation coefficient analysis (CCA) is used to evaluate the degree of linear correlation between the independent and dependent variables [21]. Its value range is [−1, 1]. A positive value means positive correlation, a negative value means negative correlation, and a larger absolute value means a higher linear correlation. In this study, the correlation coefficient between each wavelength and the SSC content was examined in order to identify the characteristic wavelengths for SSC determination. The Pearson correlation coefficient was used in this study shown as Equation (1):

(1) $$\rho_{x,y}=\frac{cov\left(x,y\right)}{\sigma_{x}\sigma_{y}}$$

where *ρ_x,y_* stands for the correlation coefficient between the variables *x* and *y*, *cov(x,y)* stands for the covariance between the variables *x* and *y*, and σ*_x_* and σ*_y_* stand for the standard deviation of *x* and *y*.

### 2.6. Model Evaluation and Software

Six indicators were used to evaluate model performance, including correlation coefficient and root mean square errors of calibration (Rc and RMSEC), correlation coefficient and root mean square errors of validation (Rv and RMSEV), and correlation coefficient and root mean square errors of prediction (Rp and RMSEP) were assessed. Generally, the closer Rc, Rv, and Rp are to 1, the higher the stability and fit of the model, while the closer RMSEC, RMSEV, and RMSEP are to 0, the stronger the predictive ability of the model is.

In this study, the ENVI 4.7 software was used to cut the image of each fruit from hyperspectral images. Matlab R2017b (The Math Works, Natick, MA, USA) software was applied to group the samples and select effective wavelengths by SPA and CCA. The SVR model was developed on PyCharm Community Edition (version 2021.2.1) (JetBrains, Prague, Czech Republic) with Python (version 3.7.12), and the PLSR model was conducted on the Unscrambler X10.1 software (CAMO AS, Oslo, Norway). Specifically, the Origin 2017 64Bit (OriginLab, Northampton, MA, USA) was used to draw spectrograms and dotted line drawings.

## 3. Results

### 3.1. Outlier Removal

In this study, 458 fruits were studied. To further establish prediction models, outliers were first identified and removed. The outliers were identified in two steps. First, since SSC value of each sample was averaged by three manual measurements. To avoid manual measurement errors, the average SSC of each fruit was first calculated, and the difference between each measurement and the average value was then calculated. Indeed, there was no particular criterion for this kind of outlier removal. In this study, when the difference between the measurement and the averaged value was larger than 15%, the sample was regarded as an outlier sample. In total, 38 samples were identified as outliers in this step. Second, the remaining 420 samples were all used to build a PLSR model, and the predictive error of each sample was calculated. In the end, 20 samples with larger predictive errors were manually identified as outliers (with a negative impact on model performances) in the second step. Next, the 400 samples were subsequently randomly divided into the calibration set (300 samples), the validation set (50 samples), and the prediction set (50 samples) at a ratio of 6:1:1. It should be noted that outlier removal was conducted using the spectra of the calyx end. As for the two hyperspectral imaging systems, the identified outlier samples were different due to the fact that the PLSR model performed differently. The statistical analysis of SSC content in different sets is shown in Table 1.

### 3.2. Spectral Profiles

**Figure 2:**
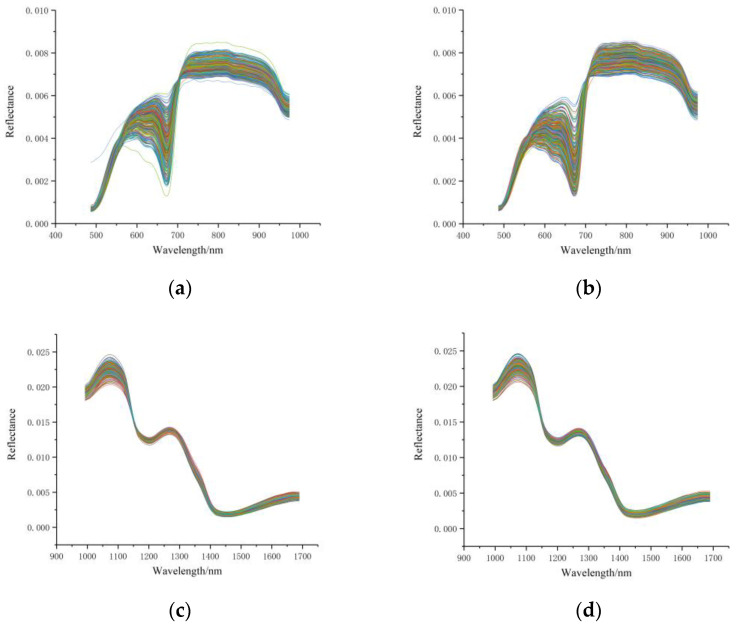
VIS/NIR spectra and NIR spectra of the fruit calyx and blossom ends. (**a**) VIS/NIR spectra of the fruit calyx end (FX10-SP-A); (**b**) VIS/NIR spectra of the fruit blossom end (FX10-SP-B); (**c**) NIR spectra of the fruit calyx end (FX17-SP-A); (**d**) NIR spectra of the fruit blossom end (FX17-SP-B).

Figure 2 shows the VIS/NIR reflectance spectra (obtained by FX10) and NIR reflectance spectra (obtained by NIR), with the horizontal coordinates indicating the 487–974 nm bands and the 933–1690 nm bands and the vertical coordinates indicating the reflectance of the spectra. Figure 2a,b shows the VIS/NIR spectra of the fruit calyx end (abbreviated as FX10-SP-A) and the fruit blossom end (abbreviated as FX10-SP-B) after removing the outlier samples. Figure 2c,d shows the spectral curves of the two sampling sides at the NIR range (FX17-SP-A and FX17-SP-B, respectively) after removing the outlier samples. It could be seen that the trends of the SP-A and SP-B curves were quite similar for each hyperspectral imaging system. From the spectral profiles, exploring the differences between the two sides for SSC measurement was difficult.

### 3.3. Regression Models

PLSR and SVR were used to establish regression models with the FX10 and FX17 datasets of the fruit calyx and blossom ends. The regression results are shown in Table 2.

For the SVR model and PLSR model, the samples were shot in the same order on the fruit calyx end and fruit blossom end. For both sides of the fruits, the samples in the calibration, validation, and prediction sets were the same. No samples were in the three sets at the same time. 

**Figure 3:**
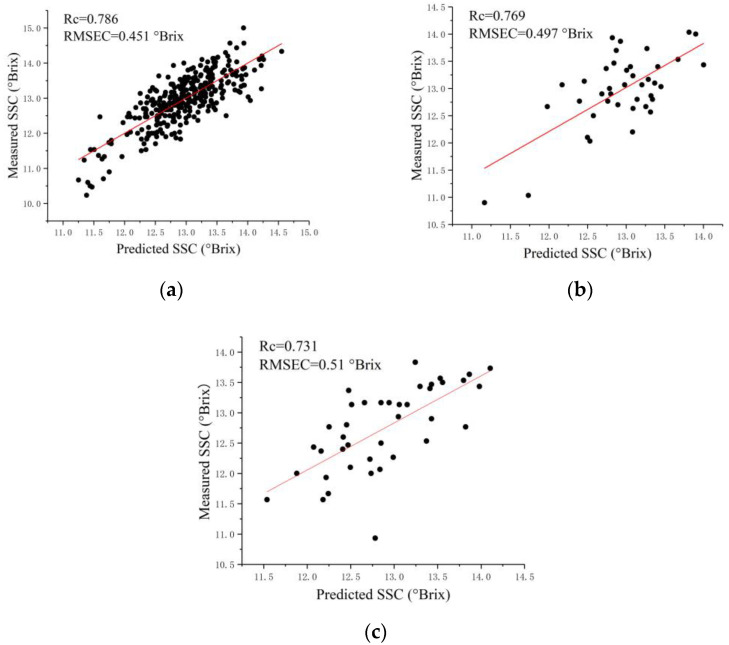
Scatter plots of the measured SSC values and predicted SSC values by SVR. (**a**) FX10-SP-A of calibration set; (**b**) FX10-SP-A of validation set; (**c**) FX10-SP-A of prediction set.

For spectra extracted from the FX10 hyperspectral images, SVR and PLSR models obtained close results for each sampling side. Regression models for the calyx end (FX10-SP-A) showed slightly better performances than those for the blossom end (FX10-SP-B), with Rc, Rv, and Rp all over 0.7, as shown in Figure 3. However, the differences in the performances between the two sides were not significant. For spectra extracted from the FX17 hyperspectral images, SVR and PLSR models also obtained close results for each sampling side. The overall prediction performances of the models for the calyx end (FX17-SP-A) were close to those for the blossom end (FX17-SP-B). The overall results of the regression models for the two sides showed differences in the performances between the sides, and the differences were insignificant. These results matched the literature [17].

It was worth investigating whether it was better to use the information of both sides instead of a single side. Thus, two different approaches to use the spectral information of the two sides were implemented. First, the average spectrum of the spectra of the two sides was calculated for each sample. Second, the low-level fusion of the spectrum of each side by directly concatenating the two spectra ranges was conducted. The results of models using average spectra and low-level fusion are shown in Table 2. For the average spectra of FX10, the PLSR model showed slightly better performances than the SVR model. For the average spectra of FX17, the SVR model showed slightly better performances than the PLSR model. For the low-level fusion of FX10, the PLSR model outperformed the SVR model. For low-level fusion of FX17, SVR, and PLSR models obtained close results.

For different datasets, the performances of the PLSR and SVR models varied. For the two sides, the performances of the same regression model were close. Moreover, the models using the average spectra of the two sides and the low-level fusion did not show significant improvement over the models using spectral profiles of a single side.

Indeed, the differences in the performances of models using a single side illustrated that there were variations of spectral profiles between the two sides. The close performances of models showed that these variations were not significant. It was a fact that both sides were in the same fruit, and the differences in spectral profiles might not be significant unless the color and maturity degree of the two sides were significantly different. When building regression models using spectra, it would be better to collect enough representative samples covering the variations of spectral profiles and SSC values. In this study, the performances of the two sides for SSC measurement did not show significant differences using different regression models, indicating no significant variations in spectral profiles. On the other hand, the two spectral ranges obtained close performances with differences, which matched the previous studies [17,22,23,24]. 

The close results of models using the spectra of a single side and the spectra of two sides (by averaging and fusing) indicated that the spectral profiles were similar within one fruit. In this study, the color and maturity degree of the two sides was close, resulting in the similarity of the spectral profiles in the visible and NIR ranges, respectively. Thus, as for the hyperspectral image, acquiring one side of the fruits might be enough to represent the sample unless the internal quality varied significantly within one fruit.

### 3.4. Analysis of Characteristic Wavelengths

The performances of models using the spectral information of a single side were close. In order to further explore the influence of spectral variations on characteristic wavelengths for SSC measurement, two variable selection methods, SPA and CCA, were used to select the characteristic wavelengths. The characteristic wavelengths of the two sides selected by SPA are shown in Table 3.

**Figure 4:**
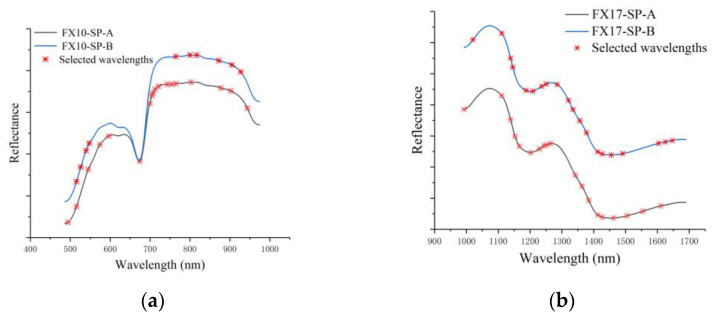
Characteristic wavelengths obtained with SPA. (**a**) Selected wavelengths of VIS/NIR spectra including SP-A and SP-B; (**b**) selected wavelengths of NIR spectra including SP-A and SP-B. (Note: Given that the average spectra of the two sides partially overlap and are not convenient for observation, the reflectance values of the SP-B spectra of FX10 were averaged and increased by 0.01, and the reflectance values of the SP-B spectra of FX17 were averaged and increased by 0.001).

As shown in Table 3, the number of selected wavelengths accounted for 10%, 6%, 19% and 20% of the total number of wavelengths at the two spectral ranges, respectively. Figure 4 shows the characteristic wavelengths selected by SPA. Although the number of characteristic wavelengths for FX10-SP-A was more than that for FX10-SP-B, they were quite similar for the selected wavelengths. The characteristic wavelengths at 516 and 765 nm of FX10 were the same for the two sides, and the characteristic wavelengths at 545, 803, 877, and 902 nm of FX10-SP-A and the characteristic wavelengths at 540, 800, 872, and 905 nm of FX10-SP-B were close. A similar phenomenon could be found for FX17 datasets. The characteristic wavelengths at 1111, 1139, 1251, 1413, and 1427 nm were the same for both sides of FX17. The characteristic wavelengths at 1202, 1230, 1342, 1462, and 1611 nm of FX17-SP-A and 1209, 1237, 1335, 1455, and 1604 nm of FX17-SP-B were quite close.

**Figure 5:**
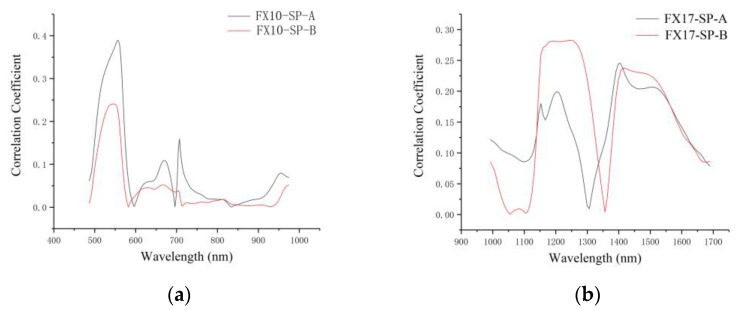
Correlation coefficient corresponding to the wavelengths. (**a**) Correlation coefficient curves of VIS/NIR averaging spectra (FX10); (**b**) correlation coefficient curves of NIR averaging spectra (FX17).

Figure 5 shows the correlation coefficient between each wavelength and the corresponding SSC values. Similarities could be found for the correlation coefficient curves of both sides. The peaks of the correlation coefficient curves of the two sides indicated the larger correlation coefficients, and these wavelengths had the potential to contribute more to SSC measurement. The wavelengths with higher correlation coefficients were 529–569 nm, 620–680 nm, 699–724 nm, and 952–974 nm for both FX10-SP-A and FX10-SP-B. For FX17, the correlation coefficient curves of both sides showed similar trends. The wavelengths at 1153–1279 nm and 1398–1547 nm showed higher correlation coefficients. 

The wavelengths in the visible range (400–780 nm) were mainly related to the color information of the fruits. The wavelength bands in the range of 780–1100 nm can be assigned as second and third overtone of C–H stretching and the second overtone of O–H stretching [25]. The wavelengths between 1200–1389 nm can be attributed to the third and second overtone of C–H stretch [25]. The wavelengths between 1400 nm and 1440 nm were related to water [26]. The wavelengths in the range of 1460–1600 nm might be related to cellulose [27]. The wavelengths between 1569 nm and 1604 nm can also be attributed to the O–H stretching of the first overtone in carbohydrates [28]. The wavelength of 1647 nm was attributed to the C–H stretching modes of aromatic C–H [29].

According to Table 3, Figure 2, Figure 4 and Figure 5, it can be seen that the spectral data of the two sides of Tribute Citru were similar, and the characteristic wavelengths that contributed more to the SSC detection were also quite similar. These results also indicated that a single side of the fruit might be enough for the SSC measurement of Tribute Citru.

SVR and PLSR were built using the characteristic wavelengths identified by SPA and CCA. The results are shown in Table 4. For characteristic wavelengths of FX10 selected by SPA, the correlation coefficients of the SVR and PLSR models of the calyx and blossom ends were around 0.6. Similar results could be observed for characteristic wavelengths of FX17 selected by SPA.

For the characteristic wavelengths of FX10 identified by the CCA method, the wavelength ranges of 529–569 nm, 620–680 nm, 699–724 nm, and 952–974 nm with similar trends of the calyx and blossom ends were selected to build PLSR and SVR models. Relatively poor performances were obtained. The correlation coefficients of the training sets of the SVR and PLSR models were below 0.7. For the characteristic wavelengths of FX17 identified by the CCA method, the bands at 1153–1279 nm and 1398–1547 nm with similar trends of the calyx and blossom ends were selected to build PLSR and SVR models. The results were not good enough.

Compared with the results in Table 3, the regression models using full range spectra performed slightly better than the corresponding models using identified characteristic wavelengths. Although characteristic wavelength selection can reduce the number of input variables and simplify the models, some useful information may also be lost.

## 4. Discussion

SSC is the most important quality attribute of concern to consumers. This study used hyperspectral imaging at two different spectral ranges to estimate the SSC in Tribute Citru, and the effect of the sampling sides of fruits was also investigated. 

The physicochemical properties within one fruit varied in different regions resulting in differences in the corresponding spectral profiles. As for point-scan near-infrared spectroscopy, spectral profiles were generally acquired from different sampling regions, and corresponding SSC values of the same sampling area were measured. Guthire et al. (2006) showed the importance of the uniformity of the spectra measurement and quality attributes measurement [30]. The influence of sampling regions has also been discussed [14,15,16]. Unlike point-scan near-infrared spectroscopy, hyperspectral imaging can acquire images of one side of the fruit facing the camera, and each pixel contains a spectrum. Based on this characteristic, HSI was generally used to acquire images of only one side of the fruits [5,6,7]. An issue should be addressed that there might also be variations of the physicochemical properties between different sides of the fruits. In this study, hyperspectral images of the two different sides of Tribute Citru fruits were acquired and compared. The overall results of the SSC prediction of the two sides showed insignificant differences using different regression methods. Further analysis of the characteristic wavelengths for SSC determination by SPA and CCA showed great similarities between the two sides. Similar results could be found in previous research, and the performances of SSC prediction using the hyperspectral images of two opposite sides of plum showed insignificant differences [17].

Two approaches to using the information of the two sides were explored, including averaging the spectra of the two sides and concatenating the spectra of the two sides. The results did not show significant improvements using the two approaches compared with models using one side. Some reasons for this might be that averaging of the spectra of the two sides could use the information of the entire fruit, and some of the variations might be reduced by averaging; concatenating the spectra of the two sides might keep the variations; and some redundant information was used, due to the fact that the spectral features of the two sides were similar. 

Relatively poor performances were obtained for the SSC determination of Tribute Citru. Some other studies have also obtained results that were not good enough [31,32,33]. Various factors affected the prediction performances (such as samples, instruments, and experimental operations, etc.), and more efforts should be made to improve the prediction performances.

This study showed that either side (the calyx and blossom ends) of the Tribute Citru could be used for SSC measurement of fruits. Although there were variations in the spectral profiles and physicochemical properties between the two sides of the Tribute Citru, future real-world applications can be developed by acquiring the hyperspectral images of either side of the fruits, which will reduce the cost of model development and online detection.

## 5. Conclusions

In this study, the influence of the sampling side of fruit for SSC determination using HSI was explored. The average of the spectra of the two sampling sides (the calyx end and the blossom end) and the fusion (direct concatenation) of the spectra of the two sampling sides were also investigated. The overall results of the two regression methods (PLSR and SVR) illustrated that the sampling sides might not significantly affect the prediction performances. Moreover, the average of the spectra of the two sampling sides and the fusion of the spectra of the two sampling sides did not significantly improve the prediction performances. The performances of the two regression methods validated the results. The characteristic wavelengths for the SSC prediction using different sampling sides by the same characteristic wavelength selection method were quite similar, indicating that sampling on either side (the calyx end and the blossom end) might be used to predict SSC. The performances of hyperspectral imaging at two different spectral ranges showed no significant differences, illustrating that both could be used for the SSC prediction of Tribute Citru. In future studies, more samples should be used to develop more robust and accurate models with machine learning methods, such as deep learning. The real-world applications of hyperspectral imaging to inspect quality and safety of Tribute Citru might then be developed in the future by scanning either side (the calyx end and the blossom end) of the samples at different spectral ranges. 

## Figures and Tables

**Table 1:** Statistical analysis of reference values of samples in the calibration, validation, and prediction sets.

Camera	Calibration Set (°Brix)			Validation Set (°Brix)			Prediction Set (°Brix)		
	Number	Min	Max	Number	Min	Max	Number	Min	Max
FX10	300	10.2	14.6	50	10.6	15.0	50	10.7	14.2
FX17	300	10.1	14.6	50	10.5	14.6	50	10.6	14.4

**Table 2:** Results and parameters of the calibration, validation, and prediction sets by SVR and PLSR models.

Dataset	Model	Model Parameter	Calibration Set		Validation Set		Prediction Set	
			Rc ^k^	RMSEC ^n^ (°Brix)	Rv ^l^	RMSEV ^o^ (°Brix)	Rp ^m^	RMSEP ^p^ (°Brix)
FX10-SP-A ^a^	SVR ^i^	Gamma: 1000.0 C: 10^6^ Eps: 10^−5^	0.810	0.435	0.750	0.511	0.705	0.53
	PLSR ^j^	Factor: 19	0.786	0.451	0.769	0.497	0.731	0.51
FX10-SP-B ^b^	SVR	Gamma: 10.0 C: 10^6^ Eps: 10^−5^	0.654	0.562	0.702	0.548	0.672	0.557
	PLSR	Factor: 16	0.744	0.487	0.705	0.558	0.655	0.571
FX17-SP-A ^c^	SVR	Gamma: 10,000.0 C: 1000.0 Eps: 10^−5^	0.725	0.497	0.744	0.578	0.588	0.567
	PLSR	Factor: 11	0.738	0.486	0.752	0.561	0.639	0.514
FX17-SP-B ^d^	SVR	Gamma: 100.0 C: 10^6^ Eps: 0.001	0.700	0.516	0.769	0.544	0.529	0.591
	PLSR	Factor: 8	0.685	0.524	0.772	0.540	0.596	0.556
FX10-average ^e^	SVR	Gamma: 100.0 C: 10^6^ Eps: 0.001	0.726	0.503	0.733	0.523	0.513	0.658
	PLSR	Factor: 17	0.782	0.455	0.751	0.515	0.736	0.515
FX17-average ^f^	SVR	Gamma: 10,000.0 C: 10^4^ Eps: 0.001	0.796	0.437	0.765	0.546	0.720	0.484
	PLSR	Factor: 11	0.747	0.479	0.773	0.540	0.611	0.540
FX10-fusion ^g^	SVR	Gamma: 1000.0 C: 10^5^ Eps: 0.001	0.882	0.344	0.707	0.566	0.529	0.675
	PLSR	Factor: 18	0.753	0.480	0.711	0.539	0.680	0.552
FX17-fusion ^h^	SVR	Gamma: 10.0 C: 10^6^ Eps: 0.0001	0.708	0.510	0.770	0.543	0.599	0.534
	PLSR	Factor: 12	0.720	0.499	0.734	0.582	0.597	0.547

^a^ FX10-SP-A: data of the calyx end obtained by FX10 camera; ^b^ FX10-SP-B: data of the blossom end obtained by FX10 camera; ^c^ FX17-SP-A: data of the calyx end obtained by FX17 camera; ^d^ FX17-SP-B: data of the blossom end obtained by FX17 camera; ^e^ FX10-average: the average spectra of the two sides obtained by FX10 camera; ^f^ FX17-average: the average spectra of the two sides obtained by FX17 camera; ^g^ FX10-fusion: the fusion spectra of the two sides obtained by FX10 camera; ^h^ FX17-fusion: the fusion spectra of the two sides obtained by FX17 camera; ^i^ SVR: support vector regression; ^j^ PLSR: partial least squares regression; ^k^ Rc: correlation coefficient of calibration; ^l^ Rv: correlation coefficient of validation; ^m^ Rp: correlation coefficient of prediction; ^n^ RMSEC: root mean square errors of calibration; ^o^ RMSEV: root mean square errors of validation; ^p^ RMSEP: root mean square errors of prediction.

**Table 3:** Characteristic wavelengths selected by SPA for the datasets of the two sides of the two hyperspectral image systems.

Datasets	Number	Characteristic Wavelengths (nm)
FX10-SP-A	18	494, 516, 545, 574, 596, 674, 699, 704, 707, 713, 721, 743, 754, 765, 803, 877, 902, 944
FX10-SP-B	10	516, 526, 540, 548, 765, 800, 817, 872, 905, 927
FX17-SP-A	19	993, 1111, 1139, 1153, 1167, 1202, 1230, 1244, 1251, 1265, 1342, 1363, 1384, 1413, 1427, 1462, 1505, 1554, 1611
FX17-SP-B	20	1020, 1111, 1139, 1146, 1188, 1209, 1237, 1251, 1286, 1321, 1335, 1356, 1377, 1413, 1427, 1455, 1490, 1604, 1626, 1647

**Table 4:** Results and parameters of the calibration, validation, and prediction sets by SVR and PLSR models using identified characteristic wavelengths.

Dataset	Model	Model Parameter	Calibration Set		Validation Set		Prediction Set	
			Rc ^k^	RMSEC ^n^ (°Brix)	Rv ^l^	RMSEV ^o^ (°Brix)	Rp ^m^	RMSEP ^p^ (°Brix)
SPA-FX10-SP-A ^a^	SVR ^i^	Gamma: 10^5^ C: 10^5^ Eps: 10^−3^	0.891	0.333	0.824	0.474	0.596	1.459
	PLSR ^j^	Factor: 15	0.771	0.465	0.767	0.495	0.678	0.544
SPA-FX10-SP-B ^b^	SVR	Gamma: 1000.0 C: 10^6^ Eps: 10^−5^	0.683	0.535	0.704	0.544	0.636	0.592
	PLSR	Factor: 9	0.704	0.518	0.675	0.564	0.664	0.568
SPA-FX17-SP-A ^c^	SVR	Gamma: 10^4^ C: 10^3^ Eps: 10^−5^	0.636	0.558	0.737	0.588	0.546	0.561
	PLSR	Factor: 14	0.743	0.482	0.718	0.592	0.670	0.498
SPA-FX17-SP-B ^d^	SVR	Gamma: 10^3^ C: 10^5^ Eps: 10^−3^	0.681	0.529	0.777	0.541	0.596	0.540
	PLSR	Factor: 8	0.689	0.522	0.774	0.537	0.594	0.559
CCA-FX10-SP-A ^e^	SVR	Gamma: 10 C: 10^6^ Eps: 10^−5^	0.571	0.603	0.686	0.560	0.456	0.650
	PLSR	Factor: 14	0.685	0.531	0.699	0.553	0.672	0.564
CCA-FX10-SP-B ^f^	SVR	Gamma: 10^3^ C: 10^5^ Eps: 10^−3^	0.667	0.550	0.604	0.609	0.663	0.560
	PLSR	Factor: 15	0.705	0.517	0.665	0.607	0.629	0.594
CCA-FX17-SP-A ^g^	SVR	Gamma: 10^5^ C: 10^3^ Eps: 10^−4^	0.714	0.507	0.794	0.533	0.555	0.565
	PLSR	Factor: 9	0.699	0.514	0.758	0.553	0.658	0.499
CCA-FX17-SP-B ^h^	SVR	Gamma: 10^4^ C: 10^5^ Eps: 10^−3^	0.697	0.517	0.778	0.534	0.519	0.626
	PLSR	Factor: 9	0.668	0.535	0.788	0.527	0.506	0.644

^a^ SPA-FX10-SP-A: data of the calyx end obtained by FX10 camera using SPA; ^b^ SPA-FX10-SP-B: data of the blossom end obtained by FX10 camera using SPA; ^c^ SPA-FX17-SP-A: data of the calyx end obtained by FX17 camera using SPA; ^d^ SPA-FX17-SP-B: data of the blossom end obtained by FX17 camera using SPA; ^e^ CCA-FX10-SP-A: data of the calyx end obtained by FX10 camera using CCA; ^f^ CCA-FX10-SP-B: data of the blossom end obtained by FX10 camera using CCA; ^g^ CCA-FX17-SP-A: data of the calyx end obtained by FX17camera using CCA; ^h^ CCA-FX17-SP-B: data of the blossom end obtained by FX17 camera using CCA; ^i^ SVR: support vector regression; ^j^ PLSR: partial least squares regression; ^k^ Rc: correlation coefficient of calibration; ^l^ Rv: correlation coefficient of validation; ^m^ Rp: correlation coefficient of prediction; ^n^ RMSEC: root mean square errors of calibration; ^o^ RMSEV: root mean square errors of validation; ^p^ RMSEP: root mean square errors of prediction.

## Data Availability

The data can be requested by contacting the corresponding authors via E-mail.
